# Effect of the macular shape on hole findings in idiopathic macular hole differs depending on the stage of the macular hole

**DOI:** 10.1038/s41598-023-42509-z

**Published:** 2023-09-16

**Authors:** Hiroto Terasaki, Toshifumi Yamashita, Ryoh Funatsu, Shohei Nomoto, Kazuki Fujiwara, Hideki Shiihara, Takehiro Yamashita, Taiji Sakamoto

**Affiliations:** Department of Ophthalmology, Kagoshima University Graduate School of Medical and Dental Sciences, Kagoshima, Japan

**Keywords:** Diseases, Medical research, Pathogenesis

## Abstract

This study aimed to investigate the relationship between macular shape and idiopathic macular hole (MH) findings using an objective method. We present retrospective observational case series on patients with MH. The shape of the macular area was quantified using quadratic equations, and the ocular shape (OS) index was calculated. The correlation between the OS index and macular hole findings for each stage was evaluated. Pearson's correlation coefficient showed a significant correlation between the OS index and horizontal hole diameter (p = 0.044), bottom diameter (p = 0.006), and vertical bottom diameter (p = 0.024) in stage 2. For stage 4, there was a negative and significant correlation between the OS index and age (p = 0.037), and horizontal (p = 0.021) and vertical (p = 0.027) bottom diameter. Multiple regression analysis showed that the horizontal (p = 0.0070) and vertical (p = 0.031) bottom diameter and OS index were independently and positively correlated in stage 2. In stage 4, the OS index was independently and negatively correlated with the horizontal (p = 0.037) and vertical (p = 0.048) bottom diameter. The ocular shape of the macula affects MH findings, and its impact depends on its stage.

## Introduction

The ocular shape is known to influence the pathogenesis of retinal and choroidal diseases. For example, in highly myopic eyes, posterior staphyloma contributes to the development of retinal separation, macular hole, etc.^1–3^. In addition, dome-shaped macula, in which the macula is convex-shaped, may result in refractory serous retinal detachment^4–6^.

Thus, the relationship between the posterior eye shape and ocular diseases has been reported mostly in highly myopic eyes. We investigated the shape of the posterior ocular region in elementary and junior high school students using optical coherence tomography (OCT) in a previous study to determine whether there are individual differences in eye shape even in the absence of myopia^7^. The results showed that variations in ocular shape exist among schoolchildren. Interestingly, in this population, approximately 20% of cases had a dome-shaped macula with the posterior eye protruding forward.

At the same time, Hirasawa et al. reported that the shape of the posterior eye correlated with the pattern of visual field abnormalities in patients with glaucoma^8^. This report indicates that the shape of the posterior segment of the eye is not only involved in the pathogenesis of high myopia, but also in non-myopic eyes.

Idiopathic macular hole (IMH) causes the circular loss of all layers of the sensory retina in the central fovea, resulting in visual loss and distorted vision^9–11^. The Gass classification, a widely used stage classification of MH, classifies the stages according to the status of the posterior vitreous detachment (PVD) and central fovea^12^. Differences in the shape of the posterior vitreous can possibly affect the force of vitreous traction in the central fovea; however, to our knowledge, no report has examined the relationship between the posterior ocular shape and IMH.

Therefore, this study examined the relationship between the posterior eye shape and IMH. In conducting the analysis, it is important to find a method to evaluate the shape of the posterior eye. For example, subjective evaluation, such as categorizing into two groups (dome and cone), has limitations in expressing differences in the degree of curvature of each group. Therefore, in this study, the curve of the posterior eye was expressed as a quadratic curve to make it a continuous variable. This study aimed to investigate the relationship between the objectively quantified shape of the macular area and morphology of the MH.

## Results

### Demographics of the patients

The demographics of the patients are shown in Table 1. A total of 59 eyes in 58 patients, 69 eyes in 69 patients, and 39 eyes in 39 patients, with a mean age of 66.8 ± 7.0, 64.9 ± 6.7, and 66.1 ± 8.5 years, respectively, were classified under MH stages 2, 3, and 4, respectively. The preoperative visual acuity (logMAR) was 0.80 ± 0.28 for stage 2, 0.72 ± 0.31 for stage 3, and 0.87 ± 0.31 for stage 4. The ocular axis length was 23.1 ± 0.97 mm in stage 2, 23.3 ± 0.99 mm in stage 3, and 23.6 ± 1.10 mm in stage 4. The ocular shape index (OSI) was 3.30 × 10^–4^ ± 0.011 in stage 2, 1.70 × 10^–4^ ± 0.023 in stage 3, and -4.90 × 10^–4^ ± 0.0024 in stage 4. The horizontal hole diameter was 439.7 ± 163.2 μm in stage 2, 369.8 ± 166.2 μm in stage 3, and 449.9 ± 182.6 μm in stage 4. The horizontal bottom diameter was 768.4 ± 255.3 μm in stage 2, 798.2 ± 280.3 μm in stage 3, and 866.5 ± 305.8 μm in stage 4. The vertical hole diameter was 380.1 ± 152.3 μm in stage 2, 351.6 ± 170.6 μm in stage 3, and 426.4 ± 186.6 m in stage 4. The vertical basal diameter was 703.3 ± 213.3 μm in stage 2, 759.4 ± 282.6 μm in stage 3, and 804.5 ± 283.8 μm in stage 4. Statistically significant differences in visual acuity (p = 0.019) and horizontal hole diameter (p = 0.015) were observed between the groups as analyzed using the Kruskal–Wallis test. Representative cases in each stage were shown in Supplemental Digital Content 1.

**Table 1:** Demographics of the patients.

	Stage 2	Stage 3	Stage 4	p value*
N (eyes)	59	69	39	–
Male/Female	24/34	25/44	10/29	0.762
Age	66.8 ± 7.0	64.9 ± 6.7	66.1 ± 8.5	0.259
Visual acuity (logMAR)	0.80 ± 0.28	0.72 ± 0.31	0.87 ± 0.31	0.019
Axial length	23.1 ± 0.97	23.3 ± 0.99	23.6 ± 1.10	0.108
Ocular shape index	3.30 × 10^–4^ ± 0.011	1.70 × 10^–4^ ± 0.023	-4.90 × 10^–4^ ± 0.0024	0.256
MH diameter horizontal	439.7 ± 163.2	369.8 ± 166.2	449.9 ± 182.6	0.015
MH basal diameter horizontall	768.4 ± 255.3	798.2 ± 280.3	866.5 ± 305.8	0.377
MH diameter vertical	380.1 ± 152.3	351.6 ± 170.6	426.4 ± 186.6	0.096
MH basal diameter vertical	703.3 ± 213.3	759.4 ± 282.6	804.5 ± 283.8	0.189

*p-value was examined using the Kruskal–Wallis test; MH, macular hole.

### Reliability of the ocular shape index measurements

Intra-rater agreement for OS index measurement was ICC 0.947 (95% CI: 0.873–0.979), and inter-rater agreement was ICC 0.933 (95% CI: 0.842–0.973), both of which were very high.

### Correlation of the ocular shape and MH parameters by stage

#### Stage 2

**Figure 1:**
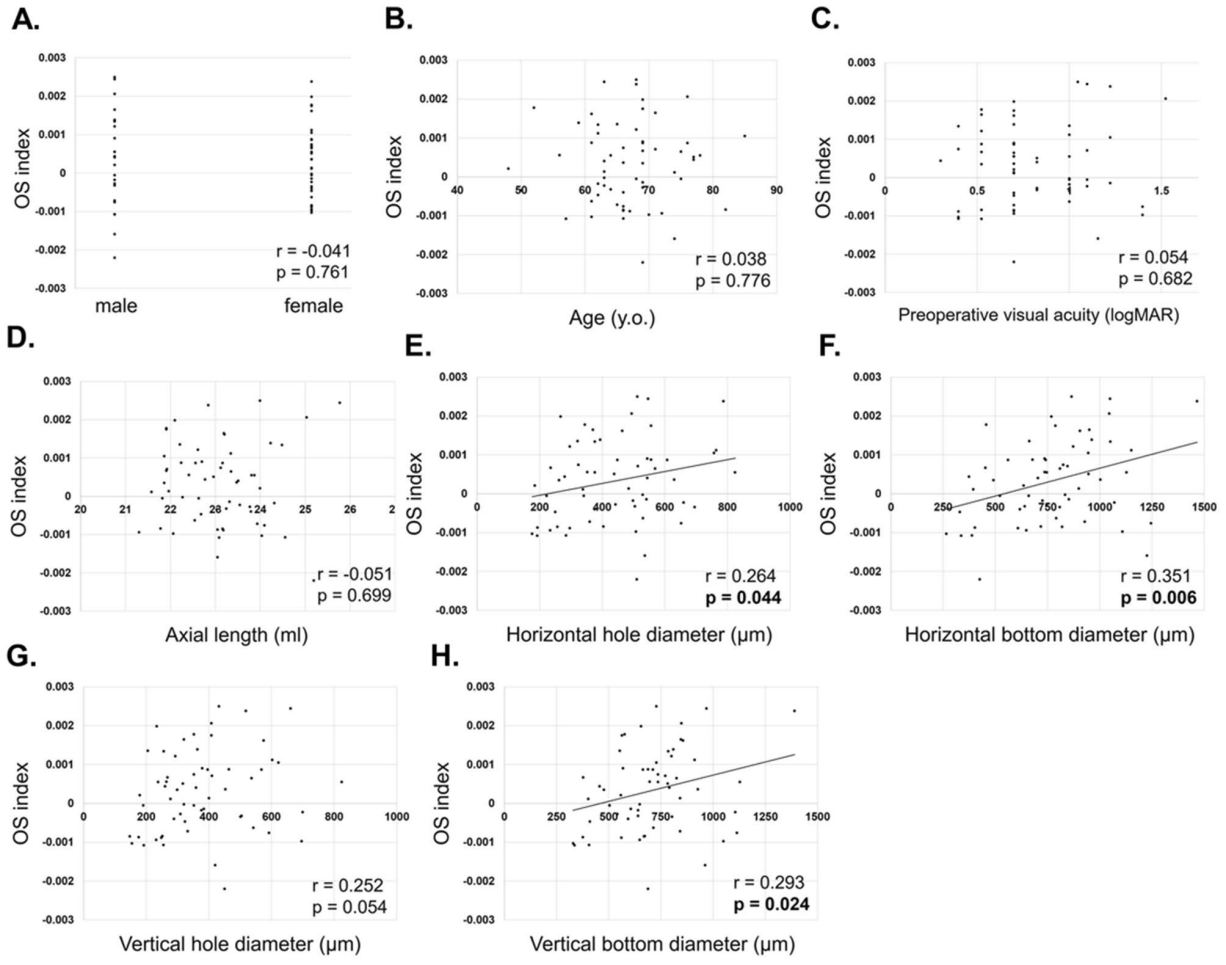
Correlation of ocular shape and macular hole parameters in stage 2. No correlation was found between ocular shape index (OSI) and gender (r = − 0.041, p = 0.761, **A**), age (r = 0.038, p = 0.776, **B**), preoperative visual acuity (r = 0.054, p = 0.682, **C**), and axial length (r = − 0.051, p = 0.699, **D**). On the other hand, there was a statistically significant correlation between the OSI and horizontal hole diameter (r = 0.264, p = 0.044, **E**), bottom diameter (r = 0.351, p = 0.006, **F**), and vertical bottom diameter (r = 0.293, p = 0.024, **H**). The OSI and vertical hole diameter tended to correlate positively but did not reach statistical significance (r = 0.252, p = 0.054, **G**).

No correlation was found between OSI and sex (r = − 0.041, p = 0.761, Fig. 1A), age (r = 0.038, p = 0.776, Fig. 1B), preoperative visual acuity (r = 0.054, p = 0.682, Fig. 1C), and axial length (r = − 0.051, p = 0.699, Fig. 1D). On the other hand, there was a statistically significant correlation between the OSI and horizontal hole diameter (r = 0.264, p = 0.044, Fig. 1E), bottom diameter (r = 0.351, p = 0.006, Fig. 1F), and vertical bottom diameter (r = 0.293, p = 0.024, Fig. 1H). The OSI and vertical hole diameter tended to correlate positively but did not reach statistical significance (r = 0.252, p = 0.054, Fig. 1G).

#### Stage 3

**Figure 2:**
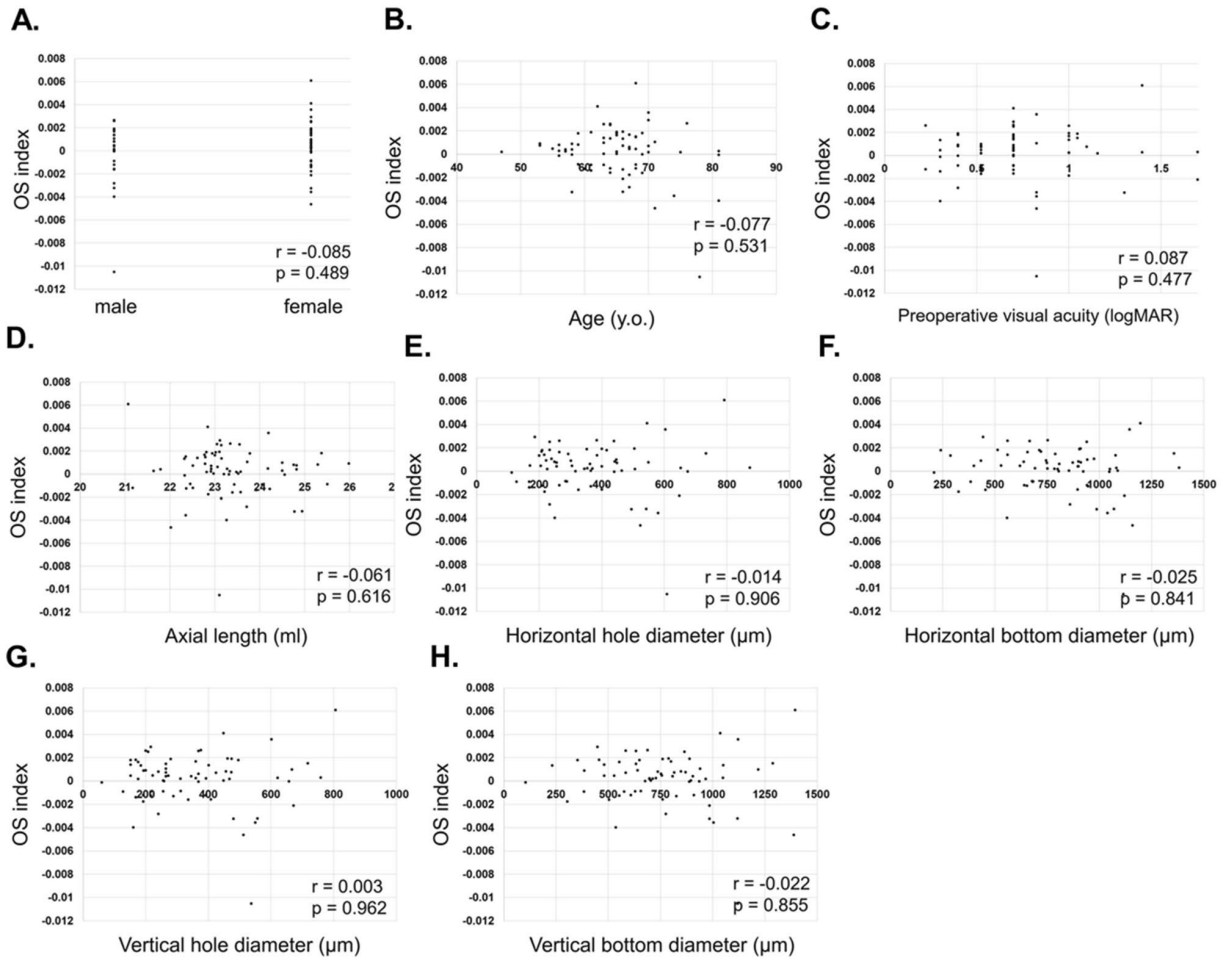
Correlation of ocular shape and macular hole parameters in stage 3. For stage 3, no correlation was found between the ocular shape index (OSI) and each factor: sex (r = − 0.085, p = 0.489, **A**), age (r = − 0.077, p = 0.531, **B**), preoperative visual acuity (r = 0.087, p = 0.477, **C**), axial length (r = − 0.0611, p = 0.616, **D**), horizontal hole diameter (r = − 0.014, p = 0.906, **E**), bottom diameter (r = − 0.025, p = 0.841, **F**), vertical hole diameter (r = 0.003, p = 0.962, **G**), bottom diameter (r = − 0. 022, p = 0.855, **H**).

For stage 3, no correlation was found between the OSI and each factor: sex (r = − 0.085, p = 0.489, Fig. 2A), age (r = − 0.077, p = 0.531, Fig. 2B), preoperative visual acuity (r = 0.087, p = 0.477, Fig. 2C), axial length (r = − 0.0611, p = 0.616, Fig. 2D), horizontal hole diameter (r = − 0.014, p = 0.906, Fig. 2E), bottom diameter (r = − 0.025, p = 0.841, Fig. 2F), vertical hole diameter (r = 0.003, p = 0.962, Fig. 2G), and bottom diameter (r = − 0.022, p = 0.855, Fig. 2H).

#### Stage 4

**Figure 3:**
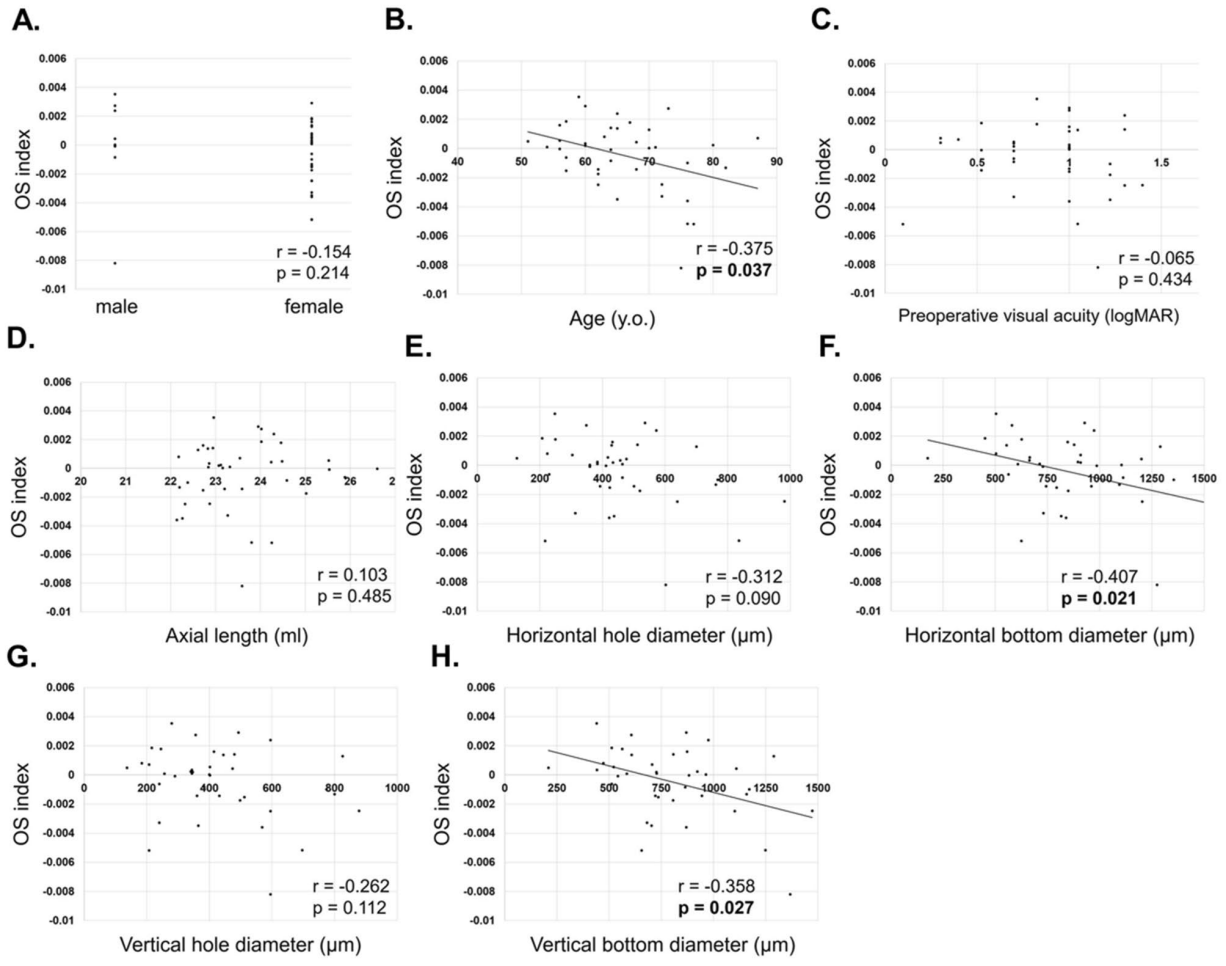
Correlation of ocular shape and macular hole parameters in stage 4. For stage 4, there was a negative and significant correlation between the ocular shape index (OSI) and age (r = − 0.375, p = 0.037, **B)** horizontal hole diameter (r = − 0.407, p = 0.021,** F**), and vertical hole diameter (r = − 0.358, p = 0.027, **H**). Other factors did not have significant difference with the OSI: sex (r = − 0.154, p = 0.214, **A**), preoperative visual acuity (r = − 0.065, p = 0.434, **C**), axial length (r = 0.103, p = 0.485, **D**), horizontal hole diameter (r = − 0.312, p = 0.09, **E**), vertical hole diameter (r = − 0.262, p = 0.112, **G**).

For stage 4, there was a negative and significant correlation between the OSI and age (r = − 0.375, p = 0.037, Fig. 3B**)**, horizontal hole diameter (r = − 0.407, p = 0.021, Fig. 3F), and vertical hole diameter (r = − 0.358, p = 0.027, Fig. 3H). Other factors did not have significant difference with OSI: sex (r = − 0.154, p = 0.214, Fig. 3A), preoperative visual acuity (r = − 0.065, p = 0.434, Fig. 3C), axial length (r = 0.103, p = 0.485, Fig. 3D), horizontal hole diameter (r = − 0.312, p = 0.09, Fig. 3E), and vertical hole diameter (r = − 0.262, p = 0.112, Fig. 3G).

### Multiple regression analysis on OSI and MH parameters

For each stage, multiple regression analysis was performed to examine the correlation between eye shape and MH parameter, using MH parameters (horizontal and vertical hole diameter and bottom diameter) as objective variables and OSI, ocular axis, and sex as explanatory variables. In stage 2, horizontal (p = 0.0070) and vertical (p = 0.031) bottom diameter and OSI were independently and positively correlated (Supplemental Digital Content 2). Stage 3 showed no significant correlation (Supplemental Digital Content 3). In Stage 4, the OSI was independently and negatively correlated with horizontal (p = 0.037) and vertical (p = 0.048) bottom diameter (Supplemental Digital Content 4).

### Macular shape correlates with visual acuity improvement at the postoperative 3rd month

Since the macular shape correlated with the hole parameters, we investigated how macular shape affects the improvement in postoperative visual acuity. While there was no correlation between the macular shape and visual acuity improvement in stages 2 and 3 (Stage 2: r = − 0.194, p = 0.164, Supplemental Digital Content 5A, Stage 3: r = − 0.089, p = 0.474, Supplemental Digital Content 5B), the improvement in visual acuity was significantly worse for dome-shaped cases in stage 4 (r = − 0.319, p = 0.048, Fig. 5C). However, multiple regression analysis with the OSI and bottom diameter as explanatory variables did not have statistical significance (Supplemental Digital Content 6).

## Discussion

In stage 2, the hole and bottom diameters were smaller in dome-shaped cases. In stage 4, the bottom diameter was larger in dome-shaped cases, which was in contrast with the results in stage 2. For the macular shape and bottom diameter, multiple regression analysis showed that statistically significant differences remained in both stages 2 and 4, but not in stage 3. In sum, a dome-shaped macula may reduce the condition of MH at stage 2 and aggravate it at stage 4.

**Figure 4:**
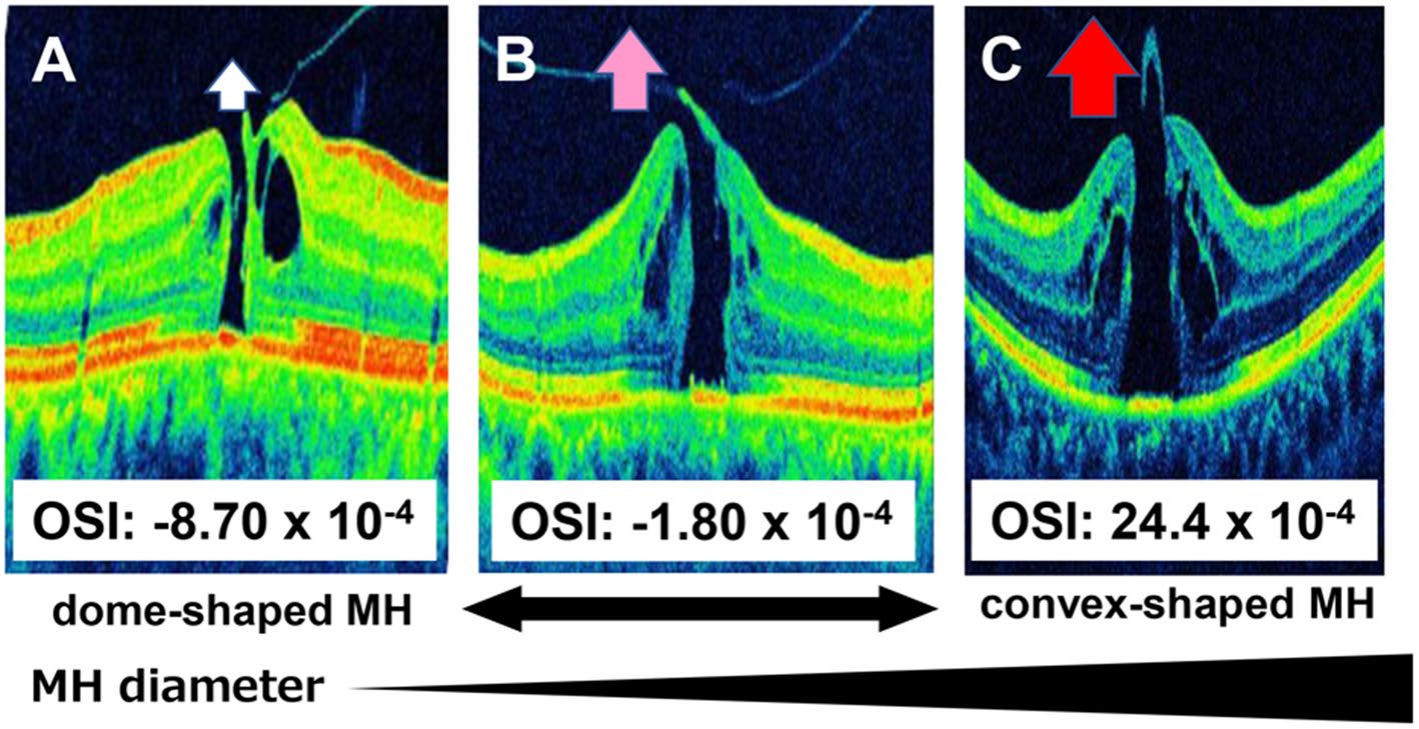
Relationship between the shape of the posterior pole and macular hole findings at stage 2. A more dome-shaped macula with an OSI of − 0.87 x 10^–4^ indicates a weaker vitreomacular traction (**A**); conversely, a more conical-shaped macula indicates a stronger vitreomacular traction (**B**, OSI = -18 × 10^–4^; **C**, OSI = 24.4 × 10^–4^).

In stage 2, unlike in stages 3 and 4, vitreous traction, which plays a major role in the enlargement of MH^13^, remains in the central fovea^12^. Screlal buckling for rhegmatogenous retinal detachment reduces the vertical vitreous traction on the retina by internalizing the sclera^14,15^. If the posterior pole is dome-shaped, the vitreous traction force on the central fovea can be weakened because of the effect similar with that of buckling compared to a cone-shaped case^14^. Thus, the present results in stage 2 are reasonable from a physical point of view (Fig. 4).

**Figure 5:**
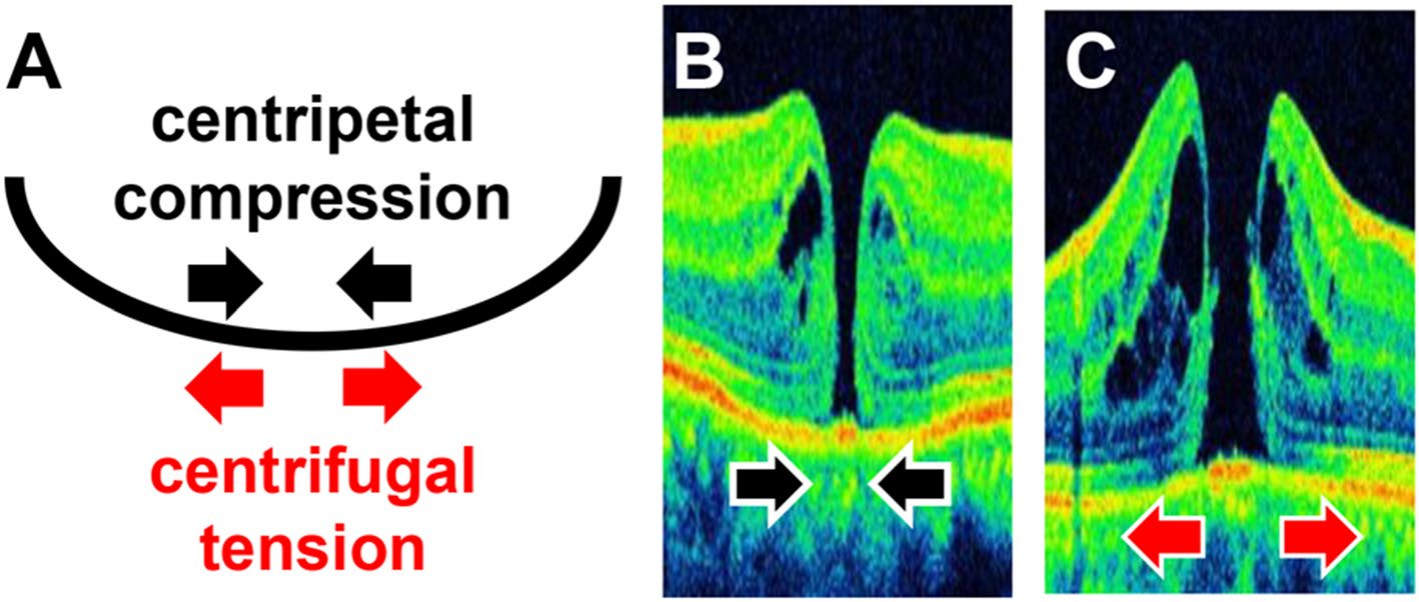
Bending stress and MH stage 4. A bent structure exerts centripetal compression force on the inside and centrifugal tension on the outside (**A**). If the macula is conical in shape, centripetal compression force is exerted on the fovea (**B**); moreover, centrifugal tension is exerted on the fovea if the macula is dome-shaped (**C**).

In stage 4, on the other hand, a statistically significant increase in the diameter of the bottom of the hole was observed with a greater dome shape. This trend is opposite with that in stage 2. In stage 4, the factor that affected MH enlargement is the tangential traction of the vitreous cortex, since there is no traction between the vitreous and central fovea^12,15^. This is known as bending stress in structural mechanics. A bent structure exerts centripetal compression forces on the inside and centrifugal tension on the outside (Fig. 5A)^16,17^. Considering this in terms of macular shape, a conical shape exerts a centripetal compression force on the fovea (Fig. 5B), while a dome shape exerts centrifugal tension on the fovea (Fig. 5C). Thus, the shape of the hole in stage 4 may be affected by bending stress due to the shape of the eye, since there is no vitreous traction. Stage 3 did not show the same correlation as stage 4, which may be due to the fact that stage 3 had a larger proportion of cases with less time since onset than stage 4^18^. In fact, preoperative visual acuity was worse in stage 4 than in stage 3 (Table 1), suggesting that more time has passed since onset in stage 4. Since the tangential traction is due to the contraction of the vitreous cortex, the ocular shape may more likely be affected in cases where PVD has occurred for a longer period of time, as in stage 4.

Reports on the effects of minor changes in anatomical location on the human body have been reported in various fields. For example, the thickness of the dermal papillary layer of the skin is approximately 200–400 μm; however, age-related flattening of the papillary layer (i.e., a change of 50–100 μm) contributes to skin fragility and pressure sores^19^. Moreover, in temporomandibular joint arthropathy, even a 1 mm deviation of the articular disk can affect symptoms^20^. In addition, irritation from misaligned dentures and implants is a well-known cause of intraoral cancer^21^. Considering these, it is reasonable that even a small change in the shape of the eyeball, which has a mean diameter of only 25 mm, can have some influence.

**Figure 6:**
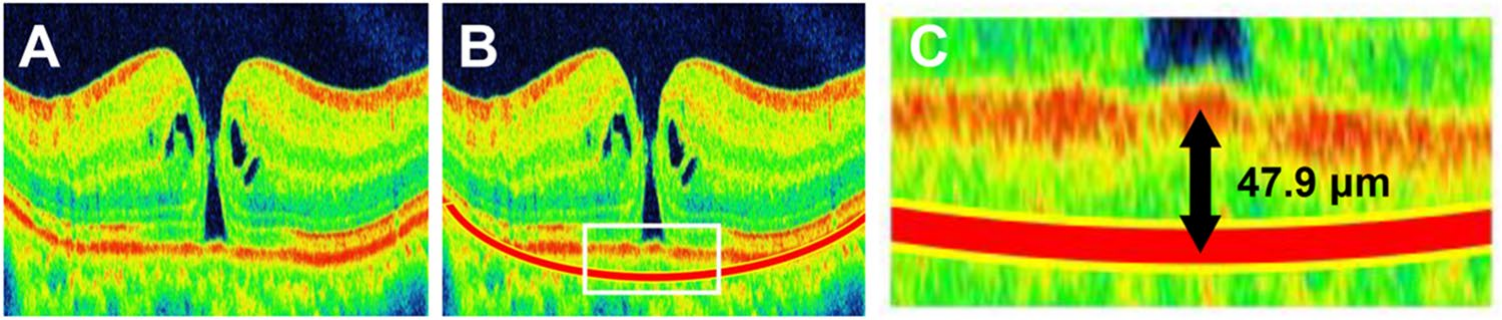
Schematic image of the effect of eye shape on the macular hole. The patient is a 63-year-old man with a stage 3 macular hole and flat macular ocular shape (**A**, ocular shape index = 0.43 × 10^–4^). If this case had a conical shape along the shape of the eye in the perimacular area (**B**, red arc line), the shift of the RPE in the fovea would be 47.9 μm in the vertical direction (measured with image J, **C**). In this case, the ocular shape index changes to 17.9 × 10^–4^.

Figure 6 is presented to examine whether small changes in the shape of the posterior pole can affect MH findings. The patient is a 63-year-old man with stage 3 MH and flat macular ocular shape (Fig. 6A, OSI = 0.43 × 10^–4^). If this case had a conical shape along the shape of the eye in the perimacular area (Fig. 6B, red arc line), the shift of the RPE in the fovea would be 47.9 μm in the vertical direction (measured with image J, Fig. 6C). This is equivalent to 13.6% of the mean vertical hole diameter of stage 3 in this study. This case also suggests that the ocular shape of the macula is a considerable factor that should not be ignored.

Although macular buckling is still performed today for refractory MH in highly myopic eyes, the rate of MH closure varies widely from 40 to 100% in the literature, with unsatisfactory results^22,23^. The results of the present study suggest that the macular buckle may be useful for retinoschisis in highly myopic eyes; however, it may have a negative effect on the closure of the hole.

The strong point of this study is that a sufficient number of cases at a single institution were included such that we were able to perform multiple regression analysis. Since it was a single-center study, the protocol for data collection, including the measurement of MH parameters, which was done subjectively, was established, and accurate data were obtained. Conversely, we acknowledge the need to examine external validity in a larger number of cases by multiple institutions in the future.

Nevertheless, this study still has some limitations including its retrospective nature and inclusion of only Japanese (all Asian) participants. Thus, further research is warranted to determine the effect of ocular shape on the pathogenesis of MH in other racial groups and different facilities. Another possibility was the limitation of our objective evaluation of ocular shape. While we obtained the degree of the ocular shape using continuous numbers, allowing for refined statistical analysis compared to subjective methods, it is essential to note that there is a large variation of ocular shape even among healthy eyes^7^. Therefore, an improved method for evaluating ocular shape might be needed. The other crucial aspect of this study is the evaluation of macular shape using vertical OCT images. As previously reported, we have observed individual differences in macular shape through the analysis of vertical OCT images in school children^7^. Additionally, our earlier research demonstrated a correlation between ocular shape and visual field defects in glaucoma, which was also based on the use of vertical OCT images^8^. Moreover, the literature supports the notion that detecting dome-shaped macula, a prevalent abnormality in ocular shape, is more effective with vertical OCT images than with horizontal OCT images^24–26^. Consequently, the adoption of vertical OCT images in this study was well-founded.

Nevertheless, it is important to consider that the identification of horizontal macular hole findings might potentially be influenced by the horizontal shape of the eye. Further investigations are warranted to address this aspect adequately, offering an opportunity for future studies to delve into this specific area.

In conclusion, we report on the impact of the ocular shape on the pathogenesis of MH. We found that the effect of the macular shape on MH differs depending on the stage of MH. In patients with MH, evaluation of the shape of the macula, in addition to the findings of the hole itself, may be useful in predicting visual prognosis and understanding the pathophysiology of MH.

## Methods

### Ethics statement

All of the procedures performed in this study conformed to the tenets of the Declaration of Helsinki and approved by the ethics committee of Kagoshima University Hospital. Informed consent was waived by ethics committee of Kagoshima University Hospital because of the retrospective nature of this study. An opportunity to opt out from the registry was provided for patients by disclosing the details of the study on the homepage of Kagoshima University Hospital.

### Participants

This was a retrospective cross-sectional study. The included cases were those of patients who received surgery at the Kagoshima University Hospital, Kagoshima, Japan from October 2012 to September 2020. The stage of IMH was determined using spectral domain OCT.

Eyes with non-IMH (e.g., MH secondary to uveitis and traumatic MH), recurrent surgery, and retinal or optic nerve diseases other than IMH were excluded.

### Examination methods

All of the eyes had a comprehensive ocular examination which included slit-lamp examinations of the anterior segment of the eye and ophthalmoscopic examinations of the fundus. The intraocular pressure (IOP) was measured with a pneumotonometer (CT-80, Topcon, Tokyo, Japan), and the axial length (AL) was measured using partial coherence laser interferometry (OA-1000, TOMEY cooperation, Nagoya, Japan). The best-corrected visual acuity (BCVA) was measured after determining the refractive error with an Auto Kerato-Refractometer (RM8900, Topcon).

### Analysis for MH findings with OCT images

OCT imaging was performed using CIRRUS OCT (Carl Zeiss Meditec Inc., Dublin, CA). The MH stage was classified by two retina specialists (HT, TY) based on the Gass grading system^12^.

In the horizontal and vertical OCT B-scan of the MH, the scan interval was set to 0.125 mm or less, and the hole diameter was measured using the caliper attached to the OCT. The hole diameter was defined as the distance with the largest hole diameter in the middle of the hole. The bottom diameter was defined as the distance with the largest diameter in the bottom of the hole which align with the retinal pigment epithelium (RPE).

### Evaluation of the macular shape

**Figure 7:**
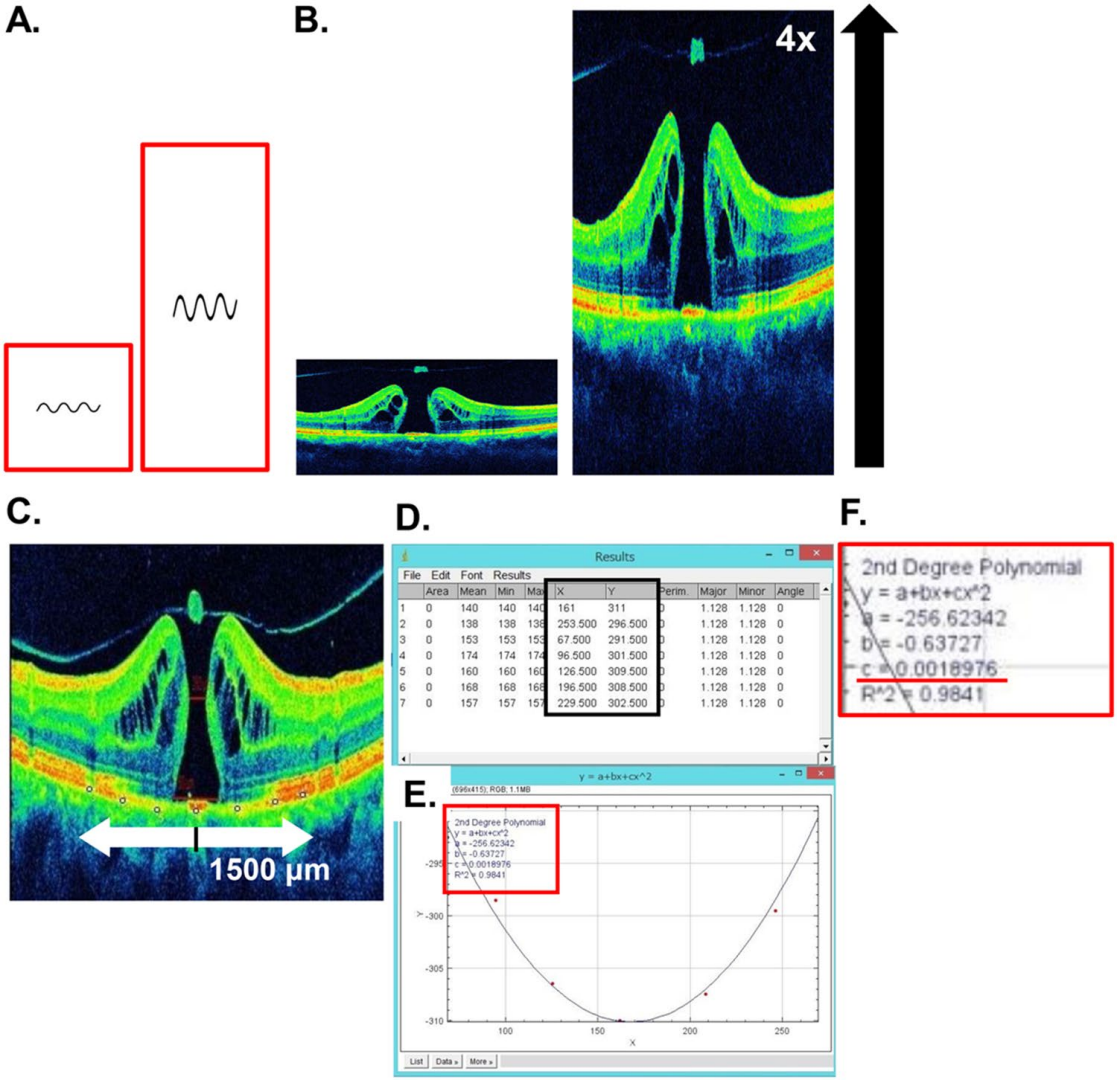
Evaluation of posterior eye shape using optical coherence tomography (OCT) images. By magnifying the image vertically, the vertical changes can be emphasized (**A**). Therefore, to evaluate the shape of the posterior eye, the vertical OCT image is vertically magnified four times (**B**). The image is then imported into ImageJ (ImageJ version 1.53 k, National Institutes of Health, Bethesda, MD; http://imagej.nih.gov/ij/) and rotated such that the line of the RPE in the center of the hole is horizontal, and the line of the RPE is plotted at seven points within 1500 μm above and below the hole. (**C**) The X and Y coordinates of the plotted points is calculated (**D**), and an approximate quadratic curve is created in curve fitting mode based on the X and Y coordinate data (**E**). The coefficient of determination in the quadratic curve equation is calculated, and this is defined as the ocular shape index (**F**, underlined).

We previously evaluated the macular shape subjectively by dividing it into dome, flat, and cone^7^, but this method did not allow us to describe the degree of cone or dome accurately. In other words, cases with a slightly dome-shaped macula and those with a strongly pronounced dome-shaped macula were classified in the same “dome-shaped macula group.” Recognizing this limitation, in this study, we endeavored to express and analyze eye shape as a continuous variable. By adopting a continuous variable approach, we aimed to overcome the need for a specific threshold value in classifying eye shape. The method for evaluating the posterior macular shape is shown in Fig. 7. In a previous report that investigated the relationship between the macular shape and ocular disease^8^, OCT images of vertical reports through the fovea were used for analysis. Moreover, a dome-shaped macula was more frequently observed on vertical OCT images than on horizontal report of OCT images of the macular area^9,24–26^. Since variations in vertical morphology were more pronounced than that of horizontal morphology in our preliminary experiments, we analyzed vertical images across the fovea in the present study using the following procedure:

1. The vertical changes can be emphasized by magnifying the image vertically (Fig. 7A). Therefore, as previously reported^7,8^, the vertical OCT image was vertically quadrupled to evaluate the shape of the macular area (Fig. 7B).
2. The image was then imported into ImageJ (ImageJ version 1.53 k, National Institutes of Health, Bethesda, MD; http://imagej.nih.gov/ij/) and rotated such that the line of the RPE in the central hole is horizontal.
3. The line of the RPE was plotted at seven locations within 1500 μm above and below the center of the MH (Fig. 7C).
4. The X and Y coordinates of the plotted points were calculated (Fig. 7D), noting that the +/− of the Y-axis coordinates is reversed in the ImageJ settings (the upper side of the image has lower values).
5. Based on the X and Y coordinate data obtained in (4), an approximate curve of a quadratic curve was created in Curve fitting mode (Fig. 7E).
6. The coefficient of determination in the quadratic curve equation was calculated. This was defined as the ocular shape index (OSI) (Fig. 7F).

In this method, a smaller OSI indicates a more dome-shaped macula, while a larger OSI indicates a more conical macula.

Inter-rate and intra-rater agreement were examined using 20 randomly selected eyes from the present case to evaluate the reproducibility of this method.

### Correlation between OSI and MH parameters

Correlations between the OSI and MH parameters (vertical and horizontal central and basal diameters of the hole) were examined using Pearson's correlation coefficient for each stage of MH. Furthermore, multiple regression analysis was performed to examine the correlation between the ocular and MH shape for each stage, using MH parameters (horizontal and vertical central and basal hole diameters) as objective variables and OSI, ocular axis and sex as explanatory variables. P < 0.05 was considered statistically significant.

### Supplementary Information

Supplementary Information 1.

Supplementary Information 2.

Supplementary Information 3.

Supplementary Information 4.

Supplementary Information 5.

Supplementary Information 6.

## Data Availability

The datasets used and/or analysed during the current study available from the corresponding author on reasonable request.
