# Supplementary material for: Effect of the macular shape on hole findings in idiopathic macular hole differs depending on the stage of the macular hole
Source: Sci Rep. 2023 Sep 16;13:15367. doi: 10.1038/s41598-023-42509-z (PMC10505151; doi:10.1038/s41598-023-42509-z)
Supplement: Supplementary file 2 — Supplementary Information 2. [file 41598_2023_42509_MOESM2_ESM.docx]

**Effect of the macular shape on hole findings in idiopathic macular hole differs depending on the stage of the macular hole**

**Running head:** Effect of the macular shape on MH

Hiroto Terasaki*, Toshifumi Yamashita, Ryoh Funatsu, Shohei Nomoto, Kazuki Fujiwara, Hideki Shiihara, Takehiro Yamashita, Taiji Sakamoto

Department of Ophthalmology, Kagoshima University Graduate School of Medical and Dental Sciences, Kagoshima, Japan

|  | Hole diameter | | | | Bottom diameter | | | |
| --- | --- | --- | --- | --- | --- | --- | --- | --- |
|  | Horizontal | | Vertical | | Horizontal | | Vertical | |
|  | R | P value | R | P value | R | P value | R | P value |
|  | Adjusted R^2^ = 0.059 | | Adjusted R^2^ = 0.041 | | Adjusted R^2^ = 0.119 | | Adjusted R^2^ = 0.087 | |
| Sex | 74.5 | 0.31 | 72.4 | 0.15 | 43.14 | 0.59 | 64.0 | 0.35 |
| Age | 3.145 | 0.16 | 1.528 | 0.60 | 4.868 | 0.30 | 2.654 | 0.50 |
| Axial length, mm | 53.252 | 0.057 | 52.113 | **0.048** | 74.457 | 0.08 | 75.664 | **0.036** |
| OS index | 37059.7 | 0.064 | 29151.5 | 0.12 | 83195.9 | **0.0070** | 55830.4 | **0.031** |

**Supplemental Digital Content 2.** Multiple regression analysis of the parameters involved in the size of the hole in the eyes at stage 2

OS index, ocular shape index
