# Supplementary material for: Effect of the macular shape on hole findings in idiopathic macular hole differs depending on the stage of the macular hole
Source: Sci Rep. 2023 Sep 16;13:15367. doi: 10.1038/s41598-023-42509-z (PMC10505151; doi:10.1038/s41598-023-42509-z)
Supplement: Supplementary file 3 — Supplementary Information 3. [file 41598_2023_42509_MOESM3_ESM.docx]

**Effect of the macular shape on hole findings in idiopathic macular hole differs depending on the stage of the macular hole**

**Running head:** Effect of the macular shape on MH

Hiroto Terasaki*, Toshifumi Yamashita, Ryoh Funatsu, Shohei Nomoto, Kazuki Fujiwara, Hideki Shiihara, Takehiro Yamashita, Taiji Sakamoto

Department of Ophthalmology, Kagoshima University Graduate School of Medical and Dental Sciences, Kagoshima, Japan

**Supplemental Digital Content 3.** Multiple regression analysis of the parameters involved in the size of the hole in the eyes at stage 3

|  | Hole diameter | | | | Bottom diameter | | | |
| --- | --- | --- | --- | --- | --- | --- | --- | --- |
|  | Horizontal | | Vertical | | Horizontal | | Vertical | |
|  | R | P value | R | P value | R | P value | R | P value |
|  | Adjusted R^2^ = 0.031 | | Adjusted R^2^ = 0.0089 | | Adjusted R^2^ = -0.031 | | Adjusted R^2^ = -0.041 | |
| Sex | 98.6 | **0.031** | 89.8 | 0.057 | 41.74 | 0.59 | 45.5 | 0.57 |
| Age | 5.272 | 0.12 | 4.52 | 0.20 | 7.728 | 0.19 | 5.851 | 0.32 |
| Axial length, mm | 7.969 | 0.72 | 6.15 | 0.79 | 3.43 | 0.93 | 14.37 | 0.71 |
| OS index | -2353.144 | 0.79 | -577.924 | 0.95 | 3526.6 | 0.82 | -4937.494 | 0.76 |

OS index, ocular shape index
