# Supplementary material for: Effect of the macular shape on hole findings in idiopathic macular hole differs depending on the stage of the macular hole
Source: Sci Rep. 2023 Sep 16;13:15367. doi: 10.1038/s41598-023-42509-z (PMC10505151; doi:10.1038/s41598-023-42509-z)
Supplement: Supplementary file 4 — Supplementary Information 4. [file 41598_2023_42509_MOESM4_ESM.docx]

**Effect of the macular shape on hole findings in idiopathic macular hole differs depending on the stage of the macular hole**

**Running head:** Effect of the macular shape on MH

Hiroto Terasaki*, Toshifumi Yamashita, Ryoh Funatsu, Shohei Nomoto, Kazuki Fujiwara, Hideki Shiihara, Takehiro Yamashita, Taiji Sakamoto

Department of Ophthalmology, Kagoshima University Graduate School of Medical and Dental Sciences, Kagoshima, Japan

**Supplemental Digital Content 4.** Multiple regression analysis of the parameters involved in the size of the hole in the eyes at stage 4

|  | Hole diameter | | | | Bottom diameter | | | |
| --- | --- | --- | --- | --- | --- | --- | --- | --- |
|  | Horizontal | | Vertical | | Horizontal | | Vertical | |
|  | R | P value | R | P value | R | P value | R | P value |
|  | Adjusted R^2^ = 0.11 | | Adjusted R^2^ = 0.094 | | Adjusted R^2^ = 0.24 | | Adjusted R^2^ = 0.26 | |
| Sex | -44.2 | 0.53 | -65.3 | 0.37 | -193.4 | 0.085 | -171.5 | 0.094 |
| Age | 3.63 | 0.35 | 3.12 | 0.43 | 9.75 | 0.11 | 9.57 | 0.085 |
| Axial length, mm | -42.6 | 0.15 | -51.3 | 0.10 | -42.8 | 0.35 | -54.7 | 0.191 |
| OS index | -20804.0 | 0.16 | -17501.6 | 0.25 | -49180.7 | **0.037** | -42115.4 | **0.048** |

OS index, ocular shape index
