# Supplementary material for: Effect of the macular shape on hole findings in idiopathic macular hole differs depending on the stage of the macular hole
Source: Sci Rep. 2023 Sep 16;13:15367. doi: 10.1038/s41598-023-42509-z (PMC10505151; doi:10.1038/s41598-023-42509-z)
Supplement: Supplementary file 5 — Supplementary Information 5. [file 41598_2023_42509_MOESM5_ESM.docx]

**Effect of the macular shape on hole findings in idiopathic macular hole differs depending on the stage of the macular hole**

**Running head:** Effect of the macular shape on MH

Hiroto Terasaki*, Toshifumi Yamashita, Ryoh Funatsu, Shohei Nomoto, Kazuki Fujiwara, Hideki Shiihara, Takehiro Yamashita, Taiji Sakamoto

Department of Ophthalmology, Kagoshima University Graduate School of Medical and Dental Sciences, Kagoshima, Japan

**
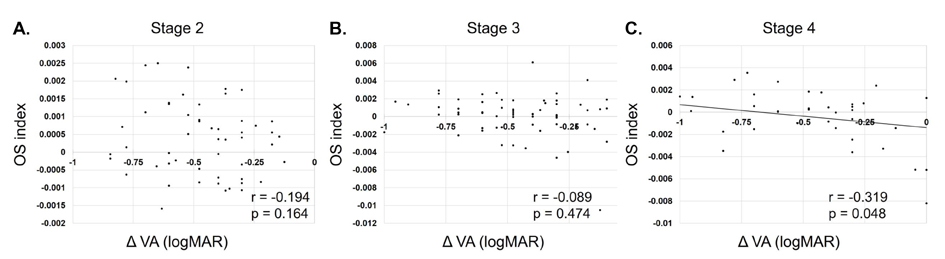
**

**Supplemental Digital Content 5. Correlationship between the macular shape and visual acuity improvement at the postoperative 3rd month**

While there was no correlation between the macular shape and visual acuity improvement in stages 2 and 3 (Stage 2: r = -0.194, P = 0.164, A, stage 3: r = -0.089, P = 0.474, B), the improvement in visual acuity was significantly worse for dome-shaped cases in stage 4 (r = -0.319, P = 0.048, C).
