# Supplementary material for: Effect of the macular shape on hole findings in idiopathic macular hole differs depending on the stage of the macular hole
Source: Sci Rep. 2023 Sep 16;13:15367. doi: 10.1038/s41598-023-42509-z (PMC10505151; doi:10.1038/s41598-023-42509-z)
Supplement: Supplementary file 6 — Supplementary Information 6. [file 41598_2023_42509_MOESM6_ESM.docx]

**Effect of the macular shape on hole findings in idiopathic macular hole differs depending on the stage of the macular hole**

**Running head:** Effect of the macular shape on MH

Hiroto Terasaki*, Toshifumi Yamashita, Ryoh Funatsu, Shohei Nomoto, Kazuki Fujiwara, Hideki Shiihara, Takehiro Yamashita, Taiji Sakamoto

Department of Ophthalmology, Kagoshima University Graduate School of Medical and Dental Sciences, Kagoshima, Japan

**Supplemental Digital Content 6. Multiple regression analysis of macular findings on changes in visual acuity at postoperative 3 months**

|  | Change in BCVA at postoperative 3 months from the preoperative period | | | | | |
| --- | --- | --- | --- | --- | --- | --- |
|  | Stage 2 | | Stage 3 | | Stage 4 | |
|  | R | P value | R | P value | R | P value |
|  | Adjusted R^2^ = 0.076 | | Adjusted R^2^ = 0.0096 | | Adjusted R^2^ = 0.11 | |
| OSI | -22.999 | 0.40 | -9.67 | 0.46 | -39.3 | 0.14 |
| Vertical Bottom diameter | -0.0002 | 0.15 | 0.000025 | 0.82 | 0.00015 | 0.50 |

BCVA, best-corrected visual acuity; OSI, ocular shape index
